# Dietary astaxanthin supplementation improves semen quality and systemic physiological health in pubertal male Nile tilapia (*Oreochromis niloticus*)

**DOI:** 10.1007/s10695-026-01677-1

**Published:** 2026-04-13

**Authors:** Patrick Gomes Avelino, Yugo Moraes Pastrana, Amanda Pereira de Amaral, Felipe Mendes de Souza, Gerlayne Maria dos Santos, Josiane Ramos da Silva, Vivian Costa Vasconcelos, Guilherme Melgaço Heluy, Romero Marcos Pedrosa Brandão Costa, Maria do Carmo Mohaupt Marques Ludke, Juliana Ferreira dos Santos, Ranilson de Souza Bezerra

**Affiliations:** 1 Enzymology Laboratory, Department of Biochemistry, Federal University of Pernambuco, Recife, PE Brazil; 2 Marine and Ornamental Fish Laboratory, Department of Aquaculture, Federal University of Santa Catarina, Florianopolis, SC Brazil; 3 Department of Fisheries and Aquaculture, Federal Rural University of Pernambuco, Recife, PE Brazil; 4 Institute of Biological Sciences, University of Pernambuco, Recife, PE Brazil; 5 Department of Animal Science, Federal Rural University of Pernambuco, Recife, PE Brazil

**Keywords:** Antioxidant capacity, Pubertal male nutrition, Fish reproduction, Hematology, Semen motility, Sperm morphology

## Abstract

This study evaluated the effects of dietary astaxanthin on sperm quality and physiological indicators of pubertal male Nile tilapia, *Oreochromis niloticus* (Linnaeus, 1758). Fish (32.42 ± 0.51 g) were allocated to 16 tanks in recirculating aquaculture system (220 L; 5 fish tank^−1^), with four replicate tanks per dietary treatment, and fed for 45 days with diets containing 0, 50, 100, and 150 mg kg^−1^ astaxanthin derived from microalgae *Haematococcus pluvialis*. Astaxanthin supplementation markedly improved seminal traits: males receiving 50–150 mg kg^−1^ exhibited significantly greater semen volume, sperm concentration and motility, and a higher proportion of morphologically normal sperm than controls (*P* < 0.05). Semen from supplemented groups also showed reduced catalase activity and malondialdehyde levels (*P* < 0.05), indicating lower oxidative stress. Growth performance and survival did not differ among treatments (*P* > 0.05). Hematologically, astaxanthin, particularly at 100–150 mg kg^−1^, increased lymphocyte proportions while decreasing circulating neutrophils, monocytes, and thrombocytes (*P* < 0.05). Serum biochemistry indicated changes in metabolic status at 100–150 mg kg^−1^, with reduced glucose, total cholesterol, triglycerides, alanine aminotransferase, and aspartate aminotransferase, and elevated total protein, albumin, and globulin (*P* < 0.05). Collectively, these findings indicate that dietary astaxanthin, particularly at 100–150 mg kg^−1^, supports semen quality and physiological status in pubertal male Nile tilapia without compromising growth.

## Introduction

Carotenoids are lipid-soluble pigments widely distributed in nature, responsible for the red, orange, and yellow coloration of plants and various animals, including fish and crustaceans (Ozogul et al. 2021). Structurally, these compounds consist of 40 carbon atoms derived from eight covalently linked isoprene units arranged in linear chains or incorporating cyclic groups at one or both ends, a molecular configuration that underlies both their pigmentation properties and potent antioxidant activity (Young and Lowe 2018; Elvira-Torales et al. 2019).

The antioxidant properties of carotenoids are central to their beneficial effects in aquaculture diets, as these compounds effectively neutralize singlet oxygen, superoxide anion radicals, and hydroxyl radicals, thereby mitigating oxidative stress through the formation of endoperoxides or epoxides (Heluy et al. 2024). Such mechanisms help prevent lipid peroxidation and offer protection against photo-oxidative damage (Maoka 2020). Among the carotenoids studied, astaxanthin stands out due to its remarkable antioxidant capacity—reported to be approximately ten times greater than other carotenoids and up to one hundred times more effective than α-tocopherol (Fang et al. 2015).

Astaxanthin is a red–orange xanthophyll carotenoid and an oxygenated derivative of carotenes that naturally occurs in microalgae (e.g., *Haematococcus pluvialis*), yeasts (*Xanthophyllomyces dendrorhous*), bacteria (*Agrobacterium aurantiacum*), crustaceans, and fish such as salmon (*Oncorhynchus* sp.) (Ambati et al. 2014; Fakhri et al. 2018). Due to its strong antioxidant potential, astaxanthin has been widely applied in cosmetics, pharmaceuticals, human dietary supplements, and as a functional feed additive in aquaculture (Higuera-Ciapara et al. 2007). In biological systems, astaxanthin exerts immunomodulatory effects by counteracting reactive oxygen species, thereby protecting hematopoietic tissues and limiting inflammatory processes (Nirmal et al. 2020). The biological efficacy of astaxanthin is influenced by its source, with *H. pluvialis* recognized as one of the richest natural sources, providing predominantly esterified astaxanthin with enhanced oxidative stability and bioavailability (Ambati et al. 2014; Zhao et al. 2023).

Fish lack the enzymatic capacity to biosynthesize astaxanthin; consequently, they rely entirely on dietary intake to obtain this carotenoid (Lim et al. 2019a, b). In this context, astaxanthin-enriched bioproducts have been incorporated into aquafeeds, resulting in improvements in body pigmentation and health indicators across several farmed fish species (Kheirabadi et al. 2022; Long et al. 2023; Heluy et al. 2024). Growing evidence also supports the role of dietary carotenoids, particularly astaxanthin, in modulating reproductive physiology in broodstock of freshwater and marine finfish and crustaceans (Ahmadi et al. 2006; Huang et al. 2008; Sawanboonchun et al. 2008; Costa et al. 2021).

In *Oreochromis* spp., however, reproductive studies involving astaxanthin have focused predominantly on females. For example, female goldfish (*Carassius auratus*) fed diets supplemented with 150 mg kg^−1^ astaxanthin produced larger eggs with higher fecundity, hatchability, and larval survival (Tizcar et al., 2015), and similar benefits have been reported in female Nile tilapia, including enhanced growth, reduced ovarian oxidative stress, and improved oocyte development (Qiang et al. 2022). In contrast, investigations addressing male reproductive physiology are largely restricted to other teleost species and typically evaluate isolated semen traits, without integrating oxidative stress biomarkers or broader physiological indicators. Consequently, information on how dietary astaxanthin affects semen quality and oxidative physiology in male Nile tilapia remains limited. This gap is particularly relevant given the increasing reliance on artificial reproduction in hatchery-based aquaculture, where male reproductive traits are key determinants of fertilization success and fry production efficiency (Bobe and Labbé, 2010; Magnotti et al. 2018).

Nile tilapia, *Oreochromis niloticus* (Linnaeus, 1758) is a freshwater species belonging to the family Cichlidae and represents the second most farmed fish species worldwide, with 5.3 million tonnes produced in 2022 (FAO 2024). The species is widely recognized for its hardiness, favorable production metrics, high consumer demand, and omnivorous feeding behavior, which facilitates the incorporation of dietary supplements such as carotenoids (El-Sayed 2020; Heluy et al. 2024).

Therefore, this study aimed to evaluate the effects of dietary inclusion of astaxanthin derived from *Haematococcus pluvialis* (0 mg kg^−1^, 50 mg kg^−1^, 100 mg kg^−1^, and 150 mg kg^−1^) at graded concentrations on semen quality parameters of pubertal male Nile tilapia. We hypothesized that astaxanthin supplementation would improve sperm quality by mitigating oxidative stress, thereby preserving membrane integrity and cellular functionality of spermatozoa. Additionally, we predicted that these effects would be dose-dependent, with moderate to high inclusion levels eliciting more pronounced physiological and seminal responses. Given the close interplay between reproductive function and systemic physiology, growth performance, hematological indicators, and serum biochemical profiles were also evaluated to provide an integrated assessment of astaxanthin’s physiological effects.

## Material and methods

### Experimental design

A total of 80 pubertal male Nile tilapia (*Oreochromis niloticus*) of the GIFT (Genetic Improvement of Farmed Tilapia) strain, obtained from the facility’s own recirculating aquaculture system, were acclimated to laboratory conditions for two weeks. Fish were sexed as males prior to the experiment but sexual maturity was not assessed at the initial body weight (32.42 ± 0.51 g). Fish were then weighed and measured (initial length = 11.00 ± 0.13 cm) and randomly distributed into 16 polyethylene tanks with a useful volume of 220 L, at a density of five fish tank^−1^. Each tank was considered one experimental unit, with four replicate tanks assigned to each dietary treatment. All tanks were connected to four independent recirculating aquaculture systems (RAS) equipped with biological and ultraviolet (UV) filtration units, ensuring continuous water treatment throughout the experimental period.

### Diets and feeding

The experimental diets were formulated based on the established nutritional composition of the ingredients and designed to meet the dietary requirements of Nile tilapia, as recommended by NRC (2011). Four diets were prepared with graded levels of astaxanthin (FormuLab® Ltd., São Paulo, Brazil): 0 mg kg^−1^ (control), 50 mg kg^−1^, 100 mg kg^−1^, and 150 mg kg^−1^ of feed (Table 1). Inclusion levels were selected based on previous studies reporting beneficial effects of astaxanthin supplementation in fish (*Oncorhynchus mykiss*) broodstock (Ahmadi et al. 2006; Tizcar et al., 2015).

**Table 1:** Ingredients and proximate composition of experimental diets (% dry matter) containing increasing levels of astaxanthin (0, 50, 100, and 150 mg kg^−1^) from *Haematococcus pluvialis* fed to pubertal male Nile tilapia for 45 days

	Astaxanthin (mg kg^−1^)			
	0	50	100	150
Ingredients (%)				
Soybean meal	63.19	63.19	63.19	63.19
Corn flour	32.59	32.59	32.59	32.59
Dicalcium phosphate	1.39	1.39	1.39	1.39
Soybean oil	0.80	0.80	0.80	0.80
Carboxymethylcellulose	0.50	0.50	0.50	0.50
Mineral-vitamin premix^a^	0.40	0.40	0.40	0.40
Limestone	0.37	0.37	0.37	0.37
DL-Methionine	0.27	0.27	0.27	0.27
L-Threonine	0.25	0.25	0.25	0.25
Salt	0.22	0.22	0.22	0.22
BHT	0.01	0.01	0.01	0.01
Inert (washed sand)	0.015	0.010	0.005	0.000
Astaxanthin	0.000	0.005	0.010	0.015
Total	100	100	100	100
Proximate composition				
Dry matter (%)	93.70	93.30	93.65	92.95
Crude protein (%)	31.29	31.41	31.20	31.36
Digestible protein (%)	28.00	28.00	28.00	28.00
Total lipids (%)	3.27	3.21	3.72	3.52
Ash (%)	5.56	5.71	5.65	5.51
Digestible energy (Kcal kg^−1^)	3200	3200	3200	3200

^a^Vit. A (2,500,000 IU); Vit. D3 (60,000 IU); Vit. E (37,500 IU); Vit. K3 (3,750 mg); Vit. B1 (4,000 mg); Vit. B2 (4,000 mg); Vit. B5 (12 g); Vit. B6 (4,000 mg); Vit. B12 (4,000 mcg); Vit. C (50 g); folic acid (1,250 mg); niacin (22.5 g); biotin (15 mg); iron (15 g); zinc (12.5 g); manganese (12.5 g); copper (2,500 mg); iodine (375 mg); cobalt (125 mg); selenium (85.7 mg)

All dry ingredients were thoroughly mixed and combined with preheated water to form a homogeneous mash. The mixture was pelleted using a laboratory extruder fitted with a 2-mm die and subsequently dried in a forced-air oven at 55 °C for 24 h. After drying, pellets were cooled to room temperature, packed in airtight containers, and stored at − 5 °C until use. No additional antioxidant compounds were added during feed preparation; however, the astaxanthin used was in its natural, predominantly esterified form, which confers enhanced oxidative stability during processing and storage.

Fish were hand-fed twice daily (10:00 and 16:00 h) for 45 days at a feeding rate of 2% of their body weight per day, and uneaten feed was collected after each feeding. Every 15 days, biometric measurements were conducted to adjust feed amounts based on biomass.

### Experimental conditions and sampling

Water temperature (29.60 ± 0.11 °C), pH (7.9 ± 0.12), and dissolved oxygen (6.60 ± 0.16 mg L^−1^) were monitored daily using a digital multiparameter probe (YSI™ Model 550 A Dissolved Oxygen Instrument, USA). Non-ionized ammonia (0.014 ± 0.03 mg L^−1^), nitrite-nitrogen (0.25 ± 0.15 mg L^−1^), and nitrate-nitrogen (5.00 ± 00 mg L^−1^) levels were measured weekly following standard procedures (Baird et al. 2017), and remained within acceptable ranges for Nile tilapia. The absence of variation in nitrate levels reflects the stability of the recirculating aquaculture system. A natural photoperiod of 12L:12D was established.

At the end of the experimental period, all fish were fasted for 24 h and anesthetized with 150 mg L^−1^ of tricaine methanesulfonate (MS-222; Sigma-Aldrich), following Araújo et al. (2018), to minimize stress during handling. Anesthesia was maintained throughout all procedures, including biometrics, blood sampling, and semen collection. Each fish was weighed on a digital balance and measured for total length using an ichthyometer. Blood samples were collected from four fish per tank (n = 16 per treatment) by caudal vein puncture using sterile syringes. Samples intended for hematological analyses were collected into tubes containing EDTA as anticoagulant, whereas samples for serum biochemical analyses were collected without anticoagulant.

The performance parameters were obtained as:

$$\begin{array}{c}\text{Final body weight }\left(\mathrm{FBW};\text{ g}\right)=\text{individually weighed at the end of the experimental period}\\ \text{Final body length }\left(\mathrm{cm}\right)=\text{individually measured at the end of the experimental period}\\ \begin{array}{c}\text{Survival rate }(\mathrm{\%}) = 100*\left(\text{final fish number}/\text{initial fish number}\right)\\ \begin{array}{c}\text{Weight gain }\left(\mathrm{WG};\text{ g}\right)=100*(\text{FBW }(\mathrm{g})-\text{initial body weight }(\mathrm{g}))/\text{initial body weight }(\mathrm{g}) \\ \text{Feed conversion ratio}=\text{dry feed fed }(\mathrm{g})/\text{WG }(\mathrm{g})\end{array}\end{array}\end{array}$$

### Semen collection and analysis

Following biometric assessment, semen was collected from each male by gentle abdominal pressure. Prior to collection, the urogenital area was carefully dried with paper towels to prevent contamination with blood, feces, urine, or mucus. The first fraction of the ejaculate was discarded to minimize potential contamination, and only the subsequent fraction was used for analyses. Semen samples were collected using dry, disposable 1-mL pipettes, replaced for each individual.

Immediately after collection, semen quality was assessed through motility, sperm concentration, and morphology:

- Sperm motility (%) = Two μL of semen were placed on a microscope slide and activated with 100 μL of distilled water. Motility was subjectively evaluated immediately after activation, within the first 20–40 s, under a light microscope (Global Optics, USA) at 10 × magnification by a single trained observer blinded to the dietary treatments. Motility duration was recorded as the time until approximately 50% of spermatozoa ceased movement. For each sample, technical replicates were evaluated, following the methodology described by Maria et al. (2006).
- Sperm concentration (cells × 10^9^ mL^−1^) = A 4 μL aliquot of semen was fixed in 996 μL of buffered formalin–saline solution. Prior to counting, samples were gently homogenized to ensure uniform cell distribution, and three subsamples were analyzed using a Neubauer hemocytometer after a short settling period.
- Sperm morphology (%) = The fixed semen sample (Hancock 1957) was diluted, and a 20 μL subsample was placed on a microscope slide and immediately stained with 5 μL of Bengal rose dye. A total of 200 sperm cells per slide were analyzed under a light microscope (Global Optics, USA) at 100 × magnification. Cells with intact head, midpiece, and tail were considered morphologically normal. Abnormal cells were classified as detached head, degenerated head, degenerated midpiece, detached tail, broken tail, coiled tail, or strongly coiled tail (Miliorini et al. 2011). The percentage of normal and abnormal spermatozoa was calculated based on the total number of observed cells.

### Antioxidant parameters

Semen samples were homogenized (40 mg mL^−1^) in 0.1 M Tris–HCl buffer containing 0.15 mM NaCl (pH 8.0) and centrifuged at 8,000 × *g* for 15 min at 4 °C. Total protein concentration in the supernatant was determined according to Bradford (1976), using bovine serum albumin as the calibration standard. Catalase (CAT) activity was quantified by monitoring the rate of hydrogen peroxide decomposition at 240 nm, at 25 °C, using a UV–visible spectrophotometer (Multiskan GO, USA) equipped with a quartz cuvette, following Aebi (1984). The results were expressed as units per milligram of protein (U mg^−1^ protein). Lipid peroxidation was assessed by determining malondialdehyde (MDA) levels using the thiobarbituric acid reactive substances (TBARS) assay, following Wallin et al. (1993). Briefly, aliquots of the samples were mixed with 7.5% trichloroacetic acid (TCA), filtered, and reacted with 0.01 M thiobarbituric acid (TBA). The reaction mixtures were incubated in a thermostatically controlled laboratory water bath at 90 °C for 45 min, and absorbance was measured at 532 nm using an ELISA-type microplate reader (Bio-Rad, USA) equipped with a fixed-wavelength absorbance filter. MDA concentrations were calculated based on calibration curves and expressed as µM MDA mg^−1^ protein.

### Hematological analyzes

Blood was drawn using disposable syringes containing 1 µL of 10% EDTA as anticoagulant. For leukocyte and thrombocyte differential counts, three blood smears were prepared per fish (48 smears per treatment) from a drop of whole blood. Slides were stained with a panoptic stain following Rosenfeld’s protocol (1947). Cell counts were performed under a light microscope (Global Optics, USA) at 40 × magnification using the indirect counting method. In this method, for each blood smear, 100 leukocytes (neutrophils, monocytes, and lymphocytes) and thrombocytes were enumerated, and the relative proportion of each cell type was calculated and expressed as a percentage (Heluy, 2024).

### Biochemical profile

Blood was pooled and centrifuged at 2,000 × *g* for 25 min at 4 °C. Serum samples were stored at − 20 °C until analysis (Jagruthi et al. 2014). Concentrations of glucose, cholesterol, triglycerides, total protein, albumin, and globulin, as well as alanine aminotransferase (ALT) and aspartate aminotransferase (AST) activities, were determined using commercial diagnostic kits (Laborlab® Ltd., Guarulhos, Brazil) in accordance with Young (1997). Spectrophotometric (Bio-Rad, USA) readings were performed strictly following the manufacturer’s instructions.

### Statistical analysis

The tank was considered the experimental unit for all analyses. Individual fish data within each tank were averaged prior to statistical testing. For serum biochemical parameters, blood samples were pooled by tank due to limited sample volume and analyzed in quadruplicate as technical replicates. Normality and homoscedasticity of tank-level data were evaluated using the Shapiro–Wilk and Levene tests, respectively. One-way analysis of variance (ANOVA) was applied using tank means as the experimental unit, and when significant differences were detected, Tukey’s post-hoc test was performed at a 5% significance level. Results are expressed as means ± standard deviation (SD) of replicate tanks. All analyses were conducted using Jamovi software, version 2.3.28 (The jamovi project, 2025).

## Results

### Semen quality

**Fig. 1:**
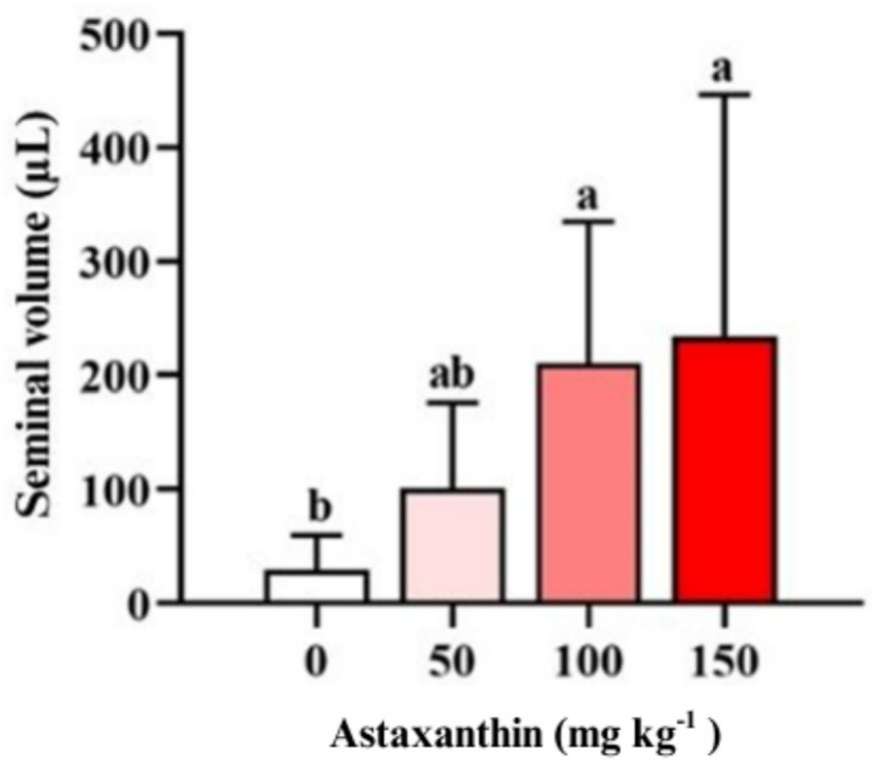
Seminal volume (µL) of Nile tilapia (*Oreochromis niloticus*) males fed diets containing increasing levels (0, 50, 100, and 150 mg kg^−1^) of astaxanthin for 45 days (*n* = 4 tanks per treatment)

Fish seminal volume increased proportionally with dietary astaxanthin concentration (Fig. 1). The control group exhibited the lowest seminal volume, averaging below 50 µL. Fish receiving 50 mg astaxanthin kg^−1^ showed an intermediate increase, reaching values above 100 μL. In contrast, males fed 100 and 150 mg kg^−1^ exhibited significantly higher seminal volumes (*P* < 0.05) than the control group.

**Fig. 2:**
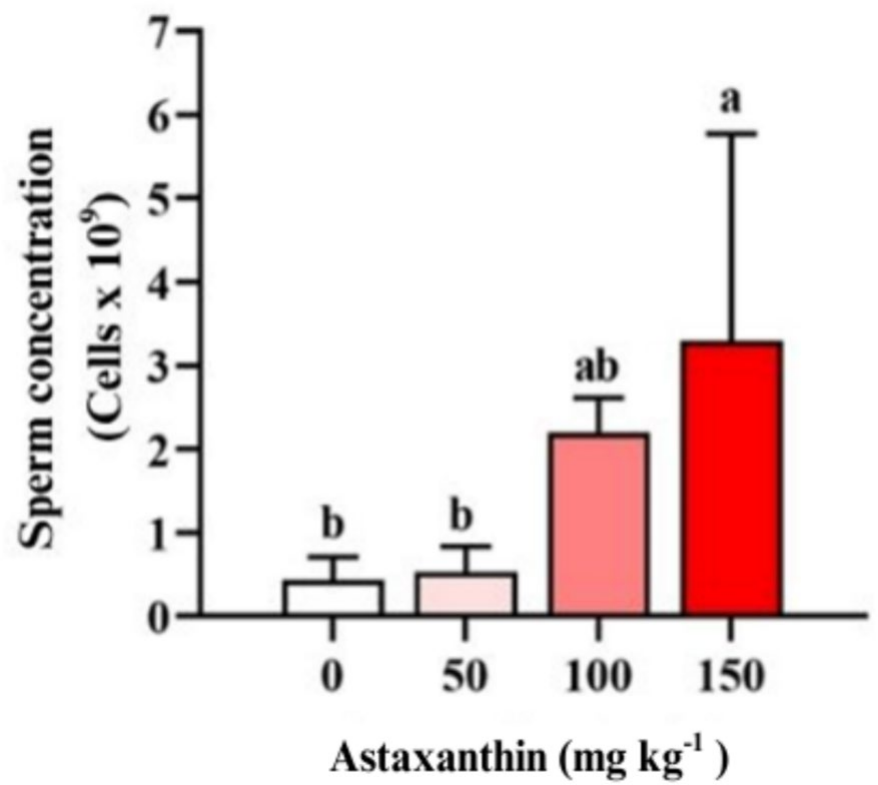
Sperm concentration (cells × 10⁹) of Nile tilapia (*Oreochromis niloticus*) males fed diets containing increasing levels (0, 50, 100, and 150 mg kg^−1^) of astaxanthin for 45 days (*n* = 4 tanks per treatment)

Sperm concentration was highest (*P* < 0.05) in males fed 150 mg astaxanthin kg^−1^, reaching 3.3 ± 2.4 × 10⁹ sperm mL^−1^, which was significantly greater than the control value of 0.44 ± 0.27 × 10⁹ sperm mL^−1^. The relatively high standard deviation observed in this treatment reflects pronounced interindividual variability, a common characteristic of seminal traits in teleost fish. The 50 mg kg^−1^ (0.50 ± 0.30 × 10⁹ sperm mL^−1^) and 100 mg kg^−1^ (2.2 ± 0.4 × 10⁹ sperm mL^−1^) treatments did not differ significantly (*P* > 0.05) from the control group (Fig. 2).

**Fig. 3:**
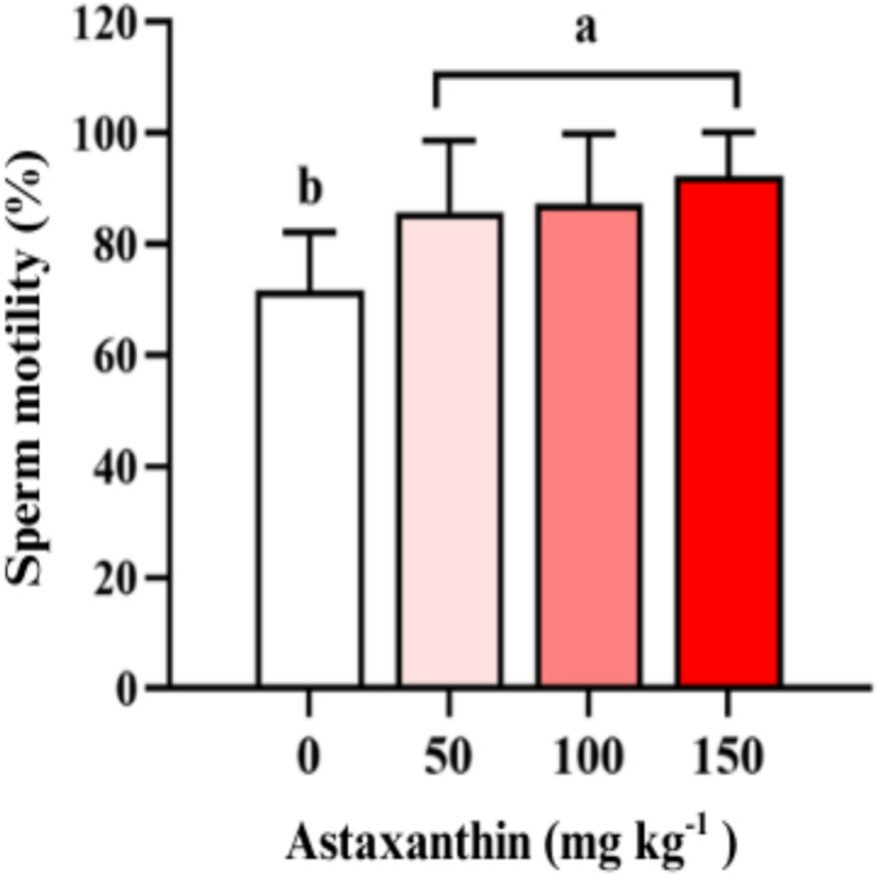
Sperm motility (%) of Nile tilapia (*Oreochromis niloticus*) males fed diets containing increasing levels (0, 50, 100, and 150 mg kg^−1^) of astaxanthin for 45 days (*n* = 4 tanks per treatment)

Astaxanthin supplementation also had a significant positive effect (*P* < 0.05) on sperm motility (Fig. 3). Fish fed 50, 100, and 150 mg kg^−1^ diets displayed motilities of 86 ± 13%, 87 ± 13%, and 92 ± 8%, respectively, representing an average increase of 17 percentage points compared with the control group (71 ± 11%).

Sperm morphology was significantly affected (*P* < 0.05) only in the percentage of morphologically normal sperm and isolated heads (CI) (Table 2). The highest proportion of normal sperm (76 ± 2%) occurred in the 100 mg kg^−1^ group, significantly exceeding the control (67 ± 6%). For CI, the lowest value (3 ± 1%) was recorded in the 100 mg kg^−1^ group, compared with 5 ± 2% (50 mg kg^−1^), 4 ± 3% (150 mg kg^−1^), and 9 ± 5% (control). No significant differences (*P* > 0.05) were detected among treatments for other abnormalities (degenerate intermediate piece, curled flagellum, tightly coiled flagellum, folded scourge, and broken scourge).

**Table 2:** Sperm morphology of Nile tilapia (*Oreochromis niloticus*) males fed diets containing increasing levels of astaxanthin (0, 50, 100, and 150 mg kg^−1^) for 45 days

Variables (%)	Astaxanthin mg kg^−1^				*P*-value
	0	50	100	150	
Normal	67.00 ± 6.00^b^	74.00 ± 6.00^ab^	76.00 ± 2.00^a^	72.00 ± 2.00^ab^	0.045
IH	9.00 ± 5.00^a^	5.00 ± 2.00^ab^	3.00 ± 1.00^b^	4.00 ± 3.00^ab^	0.045
DIP	4.00 ± 1.00^a^	5.00 ± 2.00^a^	6.00 ± 2.00^a^	6.00 ± 2.00^a^	0.34
CF	7.00 ± 2.00^a^	6.00 ± 1.00^a^	6.00 ± 1.00^a^	8.00 ± 2.00^a^	0.13
TCF	6.00 ± 4.00^a^	4.00 ± 1.00^a^	3.00 ± 1.00^a^	5.00 ± 3.00^a^	0.33
FS	3.00 ± 2.00^a^	2.00 ± 2.00^a^	3.00 ± 2.00^a^	3.00 ± 1.00^a^	0.95
BS	4.00 ± 2.00^a^	2.00 ± 1.00^a^	2.00 ± 1.00^a^	2.00 ± 1.00^a^	0.19

*Normal* sperm without morphology alteration, *IH* isolated head, *DIP* degenerate intermediate piece, *CF* curled flagellum, *TCF* tightly coiled flagellum, *FS* folded scourge, *BS* broken scourge. Different letters on the same line indicate significant differences by one-way ANOVA and Tukey tests (*P* < 0.05). Values are presented as mean ± SD (*n* = 4 tanks per treatment)

**Fig. 4:**
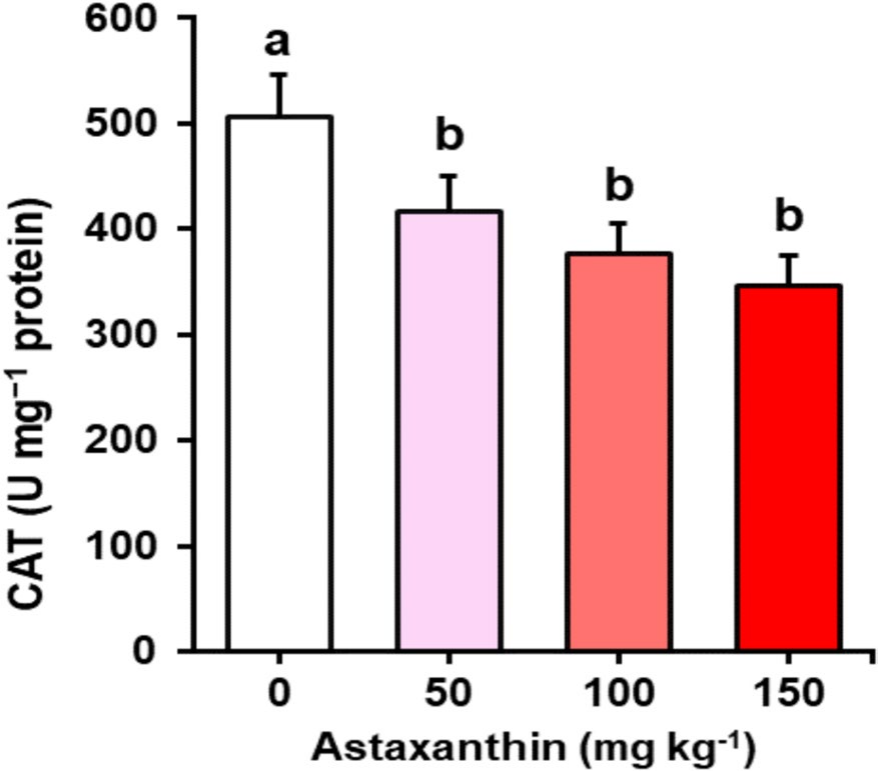
Catalase activity (U mg^−1^ protein) in sperm of Nile tilapia (*Oreochromis niloticus*) males fed diets containing increasing levels (0, 50, 100, and 150 mg kg^−1^) of astaxanthin for 45 days (*n* = 4 tanks per treatment)

The catalase (CAT) activity in sperm was significantly influenced by dietary astaxanthin (*P* = 0.002) (Fig. 4). The control group exhibited the highest enzymatic activity (> 500 U mg^−1^ protein), whereas the 50, 100, and 150 mg kg^−1^ treatments showed lower and statistically similar values (~ 400–330 U mg^−1^ protein).

**Fig. 5:**
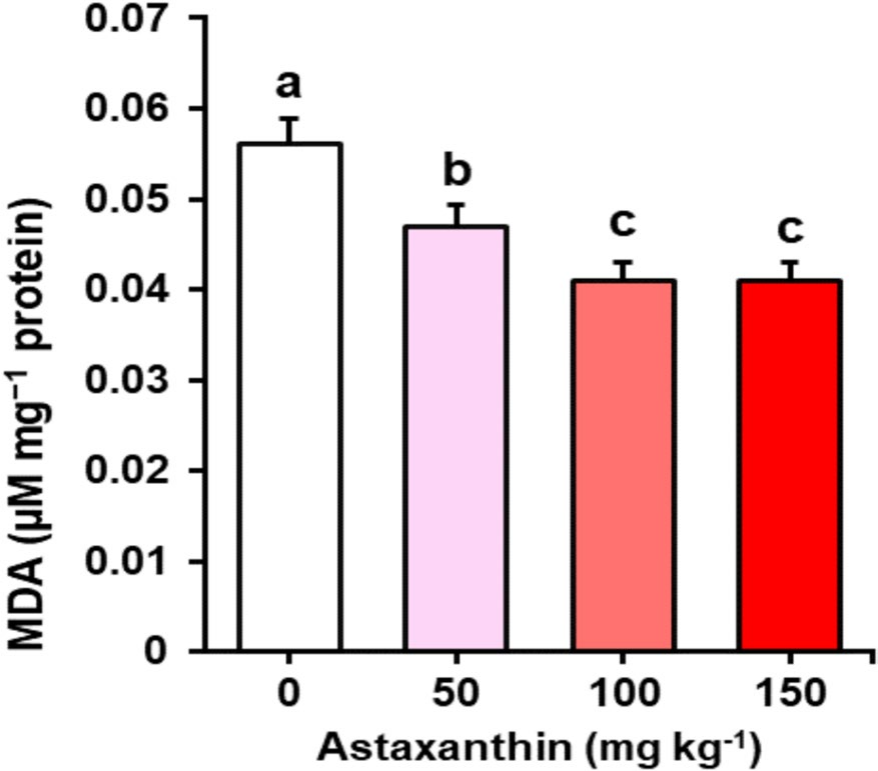
Malondialdehyde (MDA) levels (µM mg^−1^ protein) in sperm of Nile tilapia (*Oreochromis niloticus*) males fed diets containing increasing levels (0, 50, 100, and 150 mg kg^−1^) of astaxanthin for 45 days (*n* = 4 tanks per treatment)

Malondialdehyde (MDA) levels in sperm were also significantly affected (*P* < 0.001) (Fig. 5). Fish from control group recorded the highest mean value (> 0.05 µM mg^−1^ protein), while fish fed with 50, 100, and 150 mg kg^−1^ of astaxanthin in diets progressively reduced lipid peroxidation to ~ 0.05, 0.04, and 0.04 µM mg^−1^ protein, respectively. The two highest supplementation levels did not differ significantly from each other but were both significantly lower than the control.

### Growth performance

The growth performance of Nile tilapia (*Oreochromis niloticus*) pubertal males was not significantly affected (*P* > 0.05) by dietary astaxanthin supplementation (Table 3). Final weight, final length, weight gain, and feed conversion ratio remained statistically similar across all experimental groups. Survival rates were consistently high, ranging from 91.67% to 100%, with no significant differences among treatments (*P* > 0.05).

**Table 3:** Growth performance of Nile tilapia (*Oreochromis niloticus*) males fed diets containing increasing levels of astaxanthin (0, 50, 100, and 150 mg kg^−1^) for 45 days

Variables	Astaxanthin mg kg^−1^				*P*-value
	0	50	100	150	
Final weight (g)	47.01 ± 7.31^a^	48.77 ± 7.09^a^	46.23 ± 5.66^a^	45.52 ± 3.09^a^	0.54
Final length (cm)	13.34 ± 0.75^a^	13.90 ± 1.21^a^	13.55 ± 0.94^a^	13.46 ± 0.75^a^	0.41
Weight gain (g)	14.50 ± 5.81^a^	16.01 ± 2.65^a^	14.66 ± 3.11^a^	13.55 ± 2.95^a^	0.44
Feed conversion ratio	1.17 ± 0.05^a^	1.10 ± 0.14^a^	1.09 ± 0.07^a^	1.21 ± 0.15^a^	0.42
Survival (%)	100.00 ± 0.00^a^	100.00 ± 0.00^a^	91.67 ± 9.62^a^	95.83 ± 8.33^a^	0.24

Different letters on the same line indicate significant differences by one-way ANOVA and Tukey tests (*P* < 0.05). Values are presented as mean ± SD (n = 4 tanks per treatment)

### Hematological parameters

The effects of dietary astaxanthin supplementation on the hematological profile of Nile tilapia males are presented in Table 4. Neutrophil and monocyte percentages decreased significantly (*P* < 0.05) with increasing astaxanthin inclusion, with the lowest values recorded in fish fed 100 and 150 mg kg^−1^. Conversely, lymphocyte counts increased progressively with higher astaxanthin levels, reaching the highest proportion in the 150 mg kg^−1^ group (*P* < 0.05). Thrombocyte percentages also declined sharply with increasing dietary astaxanthin, with fish receiving 150 mg kg^−1^ showing the lowest counts (*P* < 0.05). These results indicate that astaxanthin supplementation modulated leukocyte distribution and markedly reduced circulating thrombocytes in Nile tilapia males.

**Table 4:** Differential leukocyte count and thrombocyte (%) of Nile tilapia (*Oreochromis niloticus*) males fed diets containing increasing levels of astaxanthin (0, 50, 100, and 150 mg kg^−1^) for 45 days

Variables (%)	Astaxanthin mg kg^−1^				*P*-value
	0	50	100	150	
Neutrophils	2.38 ± 1.01^a^	1.71 ± 0.91^a^	0.96 ± 0.86^b^	0.92 ± 0.83^b^	< 0.001
Monocytes	1.83 ± 0.87^a^	1.04 ± 0.91^b^	1.04 ± 0.75^b^	0.96 ± 0.86^b^	0.001
Lymphocytes	74.29 ± 5.44^c^	82.79 ± 6.37^b^	84.54 ± 4.15^b^	92.50 ± 2.43^a^	< 0.001
Thrombocytes	21.50 ± 5.23^a^	14.46 ± 6.16^b^	13.46 ± 3.91^b^	5.63 ± 2.34^c^	< 0.001

Different letters on the same line indicate significant differences by one-way ANOVA and Tukey tests (*P* < 0.05). Values are presented as mean ± SD (n = 4 tanks per treatment)

### Serum biochemical parameters

The serum biochemical responses of Nile tilapia males to dietary astaxanthin supplementation are shown in Table 5. Fish fed 50, 100 and 150 mg kg^−1^ astaxanthin exhibited significantly lower (*P* < 0.05) glucose, total cholesterol, triglycerides, alanine aminotransferase (ALT), and aspartate aminotransferase (AST) levels compared with the control. Albumin, total serum protein, and globulin concentrations increased significantly (*P* < 0.05) in fish receiving 150 mg kg^−1^, with intermediate values observed in the 100 mg kg^−1^ group. Overall, increasing dietary astaxanthin levels were associated with an improved lipid profile, enhanced protein status, and reduced markers of hepatic stress (ALT and AST).

**Table 5:** Biochemical serum profile of Nile tilapia (*Oreochromis niloticus*) males fed diets containing increasing levels of astaxanthin

Variables	Astaxanthin mg kg^−1^				*P*-value
	0	50	100	150	
Glucose	110.44 ± 2.24^a^	101.34 ± 1.63^b^	97.99 ± 1.98^b^	96.25 ± 0.88^b^	< 0.001
Total cholesterol	172.96 ± 4.34^a^	160.53 ± 8.73^ab^	143.20 ± 7.76^b^	142.43 ± 6.74^b^	0.003
Triglycerides	234.64 ± 11.83^a^	219.74 ± 8.68^ab^	194.79 ± 10.14^bc^	173.93 ± 7.78^c^	< 0.001
Albumin	1.91 ± 0.02^b^	1.92 ± 0.04^b^	2.02 ± 0.04^ab^	2.10 ± 0.07^a^	0.021
Total protein	4.53 ± 0.05^c^	4.65 ± 0.05^c^	5.00 ± 0.06^b^	5.40 ± 0.22^a^	< 0.001
Globulin	2.63 ± 0.06^c^	2.73 ± 0.06^bc^	2.98 ± 0.06^ab^	3.30 ± 0.22^a^	< 0.001
ALT	6.69 ± 0.66^a^	5.93 ± 0.36^ab^	4.99 ± 0.39^b^	4.65 ± 0.67^b^	0.006
AST	62.55 ± 2.52^a^	49.18 ± 3.80^b^	35.22 ± 3.81^c^	28.17 ± 1.74^c^	< 0.001

Glucose (mg dL^−1^); Total cholesterol (mg dL^−1^); Triglycerides (mg dL^−1^); Albumin (g dL^−1^); Total protein (g dL^−1^); Globulin (g dL^−1^), *ALT* alanine aminotransferase (U L^−1^), *AST* aspartate aminotransferase (U L^−1^). Different letters on the same line indicate significant differences by one-way ANOVA and Tukey tests (*P* < 0.05). Values are presented as mean ± SD (n = 4 tanks per treatment)

## Discussion

This study demonstrates that dietary astaxanthin supplementation markedly improves seminal quality in male Nile tilapia. Diets containing 100 and 150 mg kg^−1^ yielded the most pronounced enhancements, including increased seminal volume, sperm concentration, and motility, improved sperm morphology, reduced lipid peroxidation, and modulation of antioxidant enzyme activity. Comparable findings have been reported in *Carassius auratus*, in which dietary supplementation with 150 mg kg^−1^ astaxanthin for 60 days enhanced seminal osmolality, motility, fertilization rate, and sperm concentration (Tizkar et al. 2015). Similarly, Domínguez-Castanedo et al. (2015) observed increases in seminal volume, sperm concentration, and motility in *Moenkhausia sanctaefilomenae* following 90 days of dietary supplementation with natural astaxanthin derived from *Haematococcus pluvialis* (NatuRose®) at a concentration of 10 g kg^−1^ feed, attributing these improvements to the antioxidant capacity of astaxanthin and the resulting preservation of sperm quality. Morphological integrity of spermatozoa is another critical determinant of sperm functional quality (Mylonas et al. 2017; Shastak and Pelletier 2023). In the present study, astaxanthin supplementation – particularly at 100 mg kg^−1^ – was associated with a greater proportion of morphologically normal sperm and fewer isolated heads, indicating improved structural integrity of spermatozoa.

Oxidative stress is a major determinant of sperm dysfunction, impairing membrane integrity, mitochondrial activity, DNA stability, and motility (Cabrita et al. 2014). Spermatozoa are particularly vulnerable to oxidative damage due to the high content of polyunsaturated fatty acids in their plasma membranes, making redox balance a critical factor for sperm functional competence (Wang et al. 2025). In the present study, dietary astaxanthin supplementation reduced lipid peroxidation, as indicated by lower malondialdehyde levels, and decreased catalase activity in semen, reflecting a reduced oxidative burden on spermatozoa. These effects likely underpinned the observed improvements in sperm motility, morphology, and concentration, parameters that are widely used as indicators of sperm functional quality in teleost species, including *Psetta maxima* (Dreanno et al. 1999), *Cyprinus carpio* (Linhart et al. 2000), and *Oncorhynchus* spp. (Lahnsteiner 2000). Notably, the physiological responses were not strictly linear across inclusion levels, with the most consistent benefits observed at 100–150 mg kg^−1^, suggesting a threshold rather than a proportional dose–response effect. Similar antioxidant-mediated improvements in semen quality have been reported in other teleosts, such as *Pelteobagrus fulvidraco*, in which astaxanthin reduced oxidative stress biomarkers following overcrowding stress (Liu et al. 2016), and *Oncorhynchus mykiss*, where astaxanthin supplementation enhanced antioxidant defense and lowered lipid peroxidation (Shabanzadeh et al. 2023). Collectively, these findings reinforce astaxanthin’s role as an effective nutritional strategy for preserving sperm functional integrity through oxidative stress regulation.

It is important to note that dietary astaxanthin concentrations were based on formulation inclusion rates and manufacturer specifications, as no post-manufacture analytical verification was performed. Although the esterified form of astaxanthin is considered relatively stable, this represents a methodological limitation, and actual dietary concentrations may have differed from nominal values.

Although growth performance was not the primary objective of this study, the results demonstrate that, under the present experimental conditions, dietary astaxanthin supplementation at the tested inclusion levels did not adversely affect growth, feed efficiency, or fish survival. Similar outcomes have been reported in other species; for instance, Costa et al. (2022) observed no significant changes in growth performance or feed efficiency in *Lophiosilurus alexandri* broodstock supplemented with synthetic astaxanthin. Likewise, Song et al. (2017) reported no alteration in growth metrics in *Oncorhynchus mykiss* fed diets containing astaxanthin, and Yi et al. (2015) found no effect of varying dietary xanthophyll and astaxanthin ratios on growth rate or feed conversion in *Trachinotus ovatus*. This consistency across studies may be attributed to the fact that the primary role of carotenoids is associated with antioxidant activity rather than direct participation in protein or energy metabolism (Costa et al. 2022). Furthermore, all diets in the present trial were formulated to meet the established nutritional requirements for the species, thereby ensuring adequate growth and preventing potential performance impairments.

Regarding hematological parameters, neutrophils – key effectors of innate immunity involved in phagocytosis and the early inflammatory response to pathogens and tissue damage – decreased markedly from 2.38 ± 1.01 to 0.92 ± 0.83% with increasing dietary astaxanthin. In fish, changes in neutrophil counts can signal shifts in immediate immune demand and systemic inflammatory status (Witeska et al. 2021). This reduction suggests attenuation of inflammatory activation and oxidative stress, consistent with the well-documented anti-inflammatory effects of astaxanthin, which include suppression of pro-inflammatory signaling and reduction of cellular oxidative activity (Kumar et al. 2021; Li et al. 2025). Experimental studies in fish have demonstrated that dietary astaxanthin alters leukocyte profiles and tends to reduce inflammation markers (Aracati et al. 2021; Besharat et al. 2024), supporting the interpretation that the reduced neutrophil counts observed here reflect a diminished need for acute inflammatory responses.

Monocytes, precursors to macrophages and antigen-presenting cells, play critical roles in chronic inflammation, debris clearance, and coordination between innate and adaptive immunity (Uribe et al., 2011). The decline in monocyte counts observed in the present study may indicate an absence of prolonged infection episodes, likely influenced by both favorable environmental conditions and the anti-inflammatory properties of astaxanthin (Assefa and Abunna 2018; Ahmed et al. 2020). Thrombocytes, also referred to as platelets, participate in hemostasis and contribute to innate immune responses and inflammation (Köllner et al. 2004). The sharp reduction in thrombocyte counts (21.50 ± 5.23 to 5.63 ± 2.34%) following astaxanthin supplementation should be interpreted with caution. Although thrombocyte levels remained within ranges reported for healthy *Oreochromis niloticus* (Bavia et al. 2024), the observed reduction may reflect a modulation of inflammatory status under the experimental conditions, without necessarily implying improved physiological function. Similar patterns have been reported by Witeska et al. (2021), who highlighted the involvement of thrombocytes in both immune defense and coagulation processes in fish.

In contrast, lymphocytes – central effectors of adaptive immunity, responsible for specific immune responses, memory, and regulation (Cao et al. 2023) – increased from 74.29 ± 5.44 to 92.50 ± 2.43% with higher dietary astaxanthin inclusion. This shift suggests a rebalancing of the immune system: a reduction in innate inflammatory activity (lower neutrophils and monocytes) accompanied by preservation or enhancement of adaptive immunity. This pattern aligns with studies showing that astaxanthin positively modulates both specific and nonspecific immune parameters, increasing phagocytic activity, lysozyme levels, immunoglobulin production, and, in some cases, lymphocyte-to-granulocyte ratios (Lim et al. 2019a, b; Li et al. 2025).

The serum biochemical profile of Nile tilapia males fed diets containing astaxanthin revealed physiological changes consistent with the improvements observed in semen quality. Fish receiving 150 mg kg^−1^ astaxanthin exhibited significant reductions in serum glucose, total cholesterol, triglycerides, ALT, and AST, along with increases in albumin, total protein, and globulin concentrations. Collectively, these changes indicate a more balanced metabolic status and reduced physiological stress, which likely contributed to the enhancement of seminal quality. In the present study, these biochemical adjustments coincided with marked improvements in sperm motility, morphology, and concentration, suggesting that metabolic homeostasis directly supported spermatogenic efficiency and gamete viability.

Oxidative stress, resulting from an imbalance between reactive oxygen species (ROS) production and antioxidant defenses, can be mitigated by dietary astaxanthin, a carotenoid with superior antioxidant capacity that activates key redox pathways such as Nrf2 (Nuclear factor erythroid 2-related factor 2) (Shastak and Pelletier 2023). In this study, the reduction in serum glucose suggests lower metabolic stress, consistent with previous reports that astaxanthin enhances insulin sensitivity, limits hepatic glucose mobilization, and protects against oxidative liver injury (Liao et al. 2024; Zhang et al. 2024). As elevated glucose is often associated with high cortisol levels – a hallmark of chronic stress that promotes hyperglycemia and suppresses reproduction in fish (Bonga 1997; Jentoft et al. 2005; Kalinowski et al. 2019) – the observed decrease likely reflects both antioxidant action and restored metabolic–endocrine homeostasis (Besharat et al. 2024). Such regulation is particularly relevant for broodstock management, as reduced cortisol and glucose have been linked to improved spawning performance in Nile tilapia (Abu-Elala et al. 2021).

The decreases in total cholesterol and triglycerides in tilapia fed the highest astaxanthin levels indicate reduced circulating lipid accumulation. Elevated serum lipid levels and increased lipid peroxidation can impair sperm quality because sperm membranes are rich in polyunsaturated fatty acids (PUFAs) (Esmaeili et al. 2015; Ye et al. 2023). This aligns with Ciereszko and Dabrowski (1994), who reported that reduced lipid peroxidation improves membrane fluidity, benefiting the structural integrity of PUFA-rich spermatozoa. Additionally, Aracati et al. (2021) observed a significant reduction in serum triglycerides in *O. niloticus* supplemented with 200 mg kg^−1^ astaxanthin for 60 days, reinforcing the hypolipidemic potential of this carotenoid. By lowering serum lipids, astaxanthin may also indirectly enhance steroid hormone synthesis, as excessive lipid peroxidation can impair Leydig cell function and testosterone production (Abdel-Ghani et al. 2019; Li et al. 2022; Tao et al. 2024). This biochemical environment therefore favors both steroidogenesis and the maintenance of healthy spermatogenesis, as reflected in the higher proportion of morphologically normal spermatozoa in the present study.

The increases in total protein and albumin observed in astaxanthin-fed groups indicate improved nutritional status and enhanced metabolic transport capacity. Heluy et al. (2024) reported similar findings in Nile tilapia fed shrimp by-products – naturally rich in astaxanthin – observing improved serum albumin and total protein levels. Albumin plays an essential role in the transport of hormones, fatty acids, and antioxidants, as well as in osmotic regulation (Alfonso et al. 2024). Moreover, albumin binds a substantial fraction of circulating testosterone, thereby helping to maintain its bioavailable form, which is critical for reproductive tissues such as the seminiferous tubules (Porgere 2021). Similarly, globulin, which serves important immunological functions, also increased with astaxanthin supplementation. This elevation may reflect an improved immune response (Soliman et al. 2022), potentially contributing to reproductive system homeostasis. A strengthened immune status is especially relevant in intensive aquaculture systems, where broodstock are handled and may be exposed to pathogen loads; thus, dietary strategies that support physiological condition and immune function can provide substantial operational benefits.

The reductions in hepatic enzymes ALT and AST in astaxanthin-supplemented fish support the hypothesis of reduced hepatic stress and cellular damage. Besharat et al. (2024) reported that dietary astaxanthin attenuated serum ALT and AST activities in *Oncorhynchus mykiss*, while Wu et al. (2021) observed similar reductions in crucian carp (*Carassius auratus*), associated with enhanced antioxidant and immune responses and reduced liver injury. Elevated ALT and AST levels are commonly used as indicators of hepatocellular damage and oxidative stress in fish (Zaki et al. 2023), reflecting increased membrane permeability and hepatocyte necrosis. Therefore, the reductions observed in the present study reinforce the protective role of astaxanthin in hepatic health and its regulatory effect on lipid metabolism. Given that hepatic function is closely linked to vitellogenin production and the biosynthesis of reproductive hormones, a healthier liver may also contribute indirectly to the physiological conditions supporting spermatogenesis (Wallace and Bergink 1974; Tramunt et al. 2021).

An additional limitation of this study is that sexual maturity was not assessed at the beginning of the experimental period, as fish were selected based on sex but not on confirmed reproductive status at the initial body weight. Although all males produced semen at the end of the trial, indicating functional spermatogenesis, the use of fish at a pubertal stage introduces uncertainty regarding the consistency of reproductive status among individuals. This aspect should be considered when interpreting semen quality and physiological outcomes. Nevertheless, the consistency of the observed responses across multiple physiological parameters supports the robustness of the effects associated with dietary astaxanthin supplementation under the present experimental conditions.

## Conclusion

Dietary astaxanthin supplementation elicited clear improvements in sperm quality and systemic physiological status of pubertal male Nile tilapia. Inclusion levels of 100–150 mg kg^−1^ consistently enhanced semen volume, sperm concentration, motility, and morphological integrity, while reduced CAT activity and MDA levels in semen indicated lower oxidative pressure on gametes. Concurrent shifts in hematological profiles and serum biochemical markers suggest an anti-inflammatory, hepatoprotective, and metabolically favorable physiological environment. Importantly, these benefits were achieved without adverse effects on growth performance or survival.

Although direct reproductive outcomes such as fertilization rate, hatching success, and larval performance were not evaluated, the observed improvements in seminal and physiological parameters indicate that dietary astaxanthin represents a promising nutritional strategy to support semen quality and physiological condition in Nile tilapia. Future studies should validate these findings by incorporating fertilization trials, offspring performance metrics, and longer feeding periods across different broodstock ages and production conditions.

## Data Availability

The datasets generated during and/or analyzed during the current study are available from the corresponding author upon reasonable request.
